# Association of Serum Ferritin Levels and Methylprednisolone Treatment With Outcomes in Nonintubated Patients With Severe COVID-19 Pneumonia

**DOI:** 10.1001/jamanetworkopen.2021.27172

**Published:** 2021-10-04

**Authors:** Aikaterini Papamanoli, Andreas P. Kalogeropoulos, Jessica Hotelling, Jeanwoo Yoo, Prabhjot Grewal, William Predun, Robin P. Jacob, Kerry Cao, Luis A. Marcos, Hal A. Skopicki

**Affiliations:** 1 Division of Infectious Diseases, Department of Medicine, Stony Brook University, Stony Brook, New York; 2 Division of Cardiology, Department of Medicine, Stony Brook University, Stony Brook, New York; 3 Department of Medicine, Stony Brook University, Stony Brook, New York

## Abstract

**Importance:**

Serum ferritin, an acute phase marker of inflammation, has several physiologic functions, including limiting intracellular oxidative stress. Whether the effectiveness of corticosteroids differs according to serum ferritin level in COVID-19 has not been reported.

**Objective:**

To examine the association between admission serum ferritin level and methylprednisolone treatment outcomes in nonintubated patients with severe COVID-19.

**Design, Setting, and Participants:**

This retrospective cohort study included patients with severe COVID-19 admitted to an academic referral center in Stony Brook, New York, from March 1 to April 15, 2020, receiving high-flow oxygen therapy (fraction of inspired oxygen, ≥50%). The outcomes of treatment with methylprednisolone were estimated using inverse probability of treatment weights, based on a propensity score comprised of clinical and laboratory variables. Patients were followed up for 28 days. Data were analyzed from December 19, 2020, to July 22, 2021.

**Exposures:**

Systemic methylprednisolone administered per the discretion of the treating physician.

**Main Outcomes and Measures:**

The primary outcome was mortality, and the secondary outcome was a composite of death or mechanical ventilation at 28 days.

**Results:**

Among 380 patients with available ferritin data (median [IQR] age, 60 years [49-72] years; 130 [34.2%] women; 250 [65.8%] men; 310 White patients [81.6%]; 47 Black patients [12.4%]; 23 Asian patients [6.1%]), 142 patients (37.4%) received methylprednisolone (median [IQR] daily dose, 160 [120-240] mg). Ferritin levels were similar in patients who received methylprednisolone vs those who did not (median [IQR], 992 [509-1610] ng/mL vs 893 [474-1467] ng/mL; *P* = .32). In weighted analyses using tertiles of ferritin values (lower: 29-619 ng/mL; middle: 623-1316 ng/mL; upper: 1322-13 418 ng/mL), methylprednisolone was associated with lower mortality in patients with ferritin in the upper tertile (HR, 0.16; 95% CI, 0.06-0.45) and higher mortality in those with ferritin in the middle (HR, 2.46; 95% CI, 1.15-5.28) and lower (HR, 2.43; 95% CI, 1.13-5.22) tertiles (*P* for interaction < .001). Composite end point rates were lower with methylprednisolone in patients with ferritin in the upper tertile (HR, 0.45; 95% CI, 0.25-0.80) but not in those with ferritin in the middle (HR, 0.83; 95% CI, 0.50-1.39) and lower (HR, 0.89; 95% CI, 0.51-1.55) tertiles (*P* for interaction = .11).

**Conclusions and Relevance:**

In this cohort study of nonintubated patients with severe COVID-19, methylprednisolone was associated with improved clinical outcomes only among patients with admission ferritin in the upper tertile of values.

## Key Points

**Question:**

Is the acute phase protein serum ferritin an early marker associated with response to methylprednisolone in nonintubated patients with severe COVID-19 pneumonia?

**Findings:**

In this cohort study including 380 nonintubated patients with severe COVID-19 pneumonia, methylprednisolone was associated with lower mortality and reduced rates of death or mechanical ventilation only in patients with admission ferritin levels in the upper tertile of values (1322-13418 ng/mL); in contrast, there was no association with benefit among those with lower ferritin values at baseline.

**Meaning:**

These findings suggest that ferritin levels on admission may be used as a marker associated with corticosteroid response among patients with severe COVID-19 pneumonia.

## Teaser

This cohort study examines serum ferritin levels as a potential biomarker associated with response to methylprednisolone among nonintubated patients with severe COVID-19 pneumonia.

## Introduction

On January 30, 2020, the World Health Organization declared the SARS-CoV-2 outbreak a public health emergency of international concern.^1^ Treatment options for established COVID-19 are limited, with current mortality risk of 20% to 25% in hospitalized patients with severe COVID-19.^2^ Corticosteroids, initially demonstrated with low-dose dexamethasone in a large randomized clinical trial in the United Kingdom,^3^ reduced mortality in patients with severe and critical COVID-19 pneumonia. Citing favorable interim results, other randomized clinical trials with corticosteroids stopped enrollment,^4,5,6^ strengthened by a meta-analysis that followed.^7^ Specifically for methylprednisolone in COVID-19, observational work from China and the United States reported mortality benefits and reduced progression to critical illness and use of health care resources.^8,9,10,11^ However, immunophenotypic heterogeneity reflected in COVID-19 disease severity^12^ could possibly explain less positive signals from methylprednisolone use seen in recent studies.^13^

Steroids have been beneficial in targeting the inflammatory overresponse caused by SARS-CoV-2 that leads to acute distress respiratory syndrome and death but might be harmful in patients with milder disease.^3^ Could response to these agents differ according to certain biomarkers? Among biomarkers indicative of the inflammatory process in COVID-19, ferritin is associated with poor outcomes and could be used to estimate clinical worsening among patients with COVID-19, as shown in recent meta-analyses.^14,15^ Aside from its well-known role as a marker of inflammation, ferritin has also been associated with host response to infection, offering tolerance to sepsis^16^ and malaria,^17^ by limiting the intracellular oxidative stress and sequestering cytosolic iron. In animal models, ferritin knockout mice infected with mycobacterium tuberculosis had decreased survival.^18^ However, no study to date has studied the association of differential responses to steroids based on serum ferritin levels. In this retrospective cohort study of nonintubated adults with severe COVID-19 pneumonia receiving high-flow oxygen therapy, we evaluated whether admission serum ferritin could be a possible surrogate of a phenotype associated with clinical response to methylprednisolone.

## Methods

The institutional review board of Stony Brook University approved this cohort study and waived the need for informed consent because this was a retrospective study using data from medical records and no additional research procedures were performed. This report follows the Strengthening the Reporting of Observational Studies in Epidemiology (STROBE) reporting guideline for cohort studies.

### Study Population

**Figure 1.**
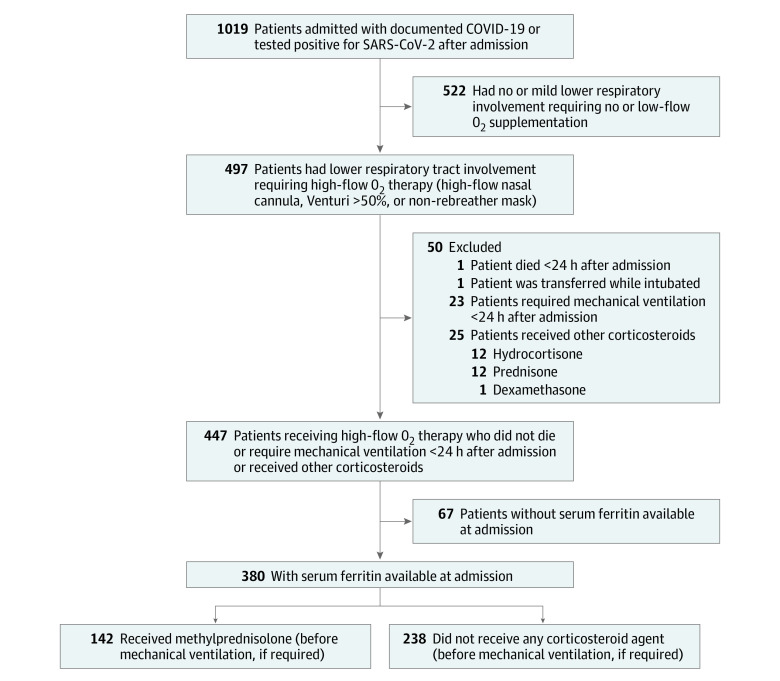
Flowchart of Study Population

We reviewed the medical records of 1019 adults (≥18 years old) admitted to Stony Brook University Hospital in Stony Brook, New York, from March 1 to April 15, 2020, with a positive result in polymerase chain reaction testing for SARS-CoV-2 and included in the study those with severe pneumonia, defined as fever or suspected respiratory infection with at least 1 of the following: respiratory rate greater than 30 breaths/min, severe respiratory distress, or oxygen saturation less than 93% with room air^19^ who required high-flow oxygen (nonrebreather mask, Venturi mask with fraction of inspired oxygen ≥50% or high-flow nasal cannula, bilevel or continuous positive airway pressure). We considered bilevel or continuous positive airway pressure as modalities of advanced oxygen therapy rather than forms of mechanical ventilation. Patients who died or required mechanical ventilation within less than 24 hours of admission, were admitted in critical condition owing to nonrespiratory causes and subsequently had test results positive for SARS-CoV-2, or received corticosteroids other than methylprednisolone were excluded (Figure 1).

### Main Exposures

Institutional guidelines during that period recommended methylprednisolone only in moderate to severe acute respiratory distress in patients undergoing mechanical ventilation, followed by slow tapering every 3 days, over 2 to 3 weeks. However, for nonintubated patients, corticosteroids were considered on a case-by-case basis, providing the opportunity to study the use of methylprednisolone in an observational setting, which was the rationale for selection of patients receiving high-flow oxygen for our study. Tocilizumab was only administered in the context of early clinical trials at that point and late during the hospital course of the patient. Additional details on data sources and corticosteroid use in our institution have been previously reported, including the association of corticosteroids use with outcomes in our population or patients receiving high-flow oxygen.^11^ Race and ethnicity were self-reported. We included race and ethnicity as covariates in the calculation of the propensity score because previous studies have reported that these are factors that associated with outcomes in patients with COVID-19. Patients who received corticosteroids only after beginning mechanical ventilation were not included in this analysis. Serum ferritin level was assayed using an electrochemiluminescence immunoassay with a Roche COBAS analyzer. Ferritin was measured during the day of admission or the next morning if admission was done overnight, and full laboratory analyses were drawn in the morning, ie, all values were within 24 hours of admission. Ferritin was available on admission in 380 of 447 patients who qualified for our study (85.0%).

### Follow-up and End Points

The primary end point in this analysis was 28-day mortality. The secondary end point was the composite of death or need for mechanical ventilation at 28 days.

Follow-up data were collected until death, hospital discharge, or readmission for COVID-19 related causes. We used the 28-day outcome framework based on the RECOVERY trial.^4^ Patients not readmitted by 28 days were considered alive and out of the hospital analysis. Patients still hospitalized by the date of database lock (July 4, 2020) were censored as alive.

### Statistical Analysis

Cox proportional hazards models were used to examine the associations among admission ferritin levels, methylprednisolone, and 28-day mortality, introducing interaction terms when appropriate. Based on the Schoenfeld residuals, there was insufficient evidence that hazard ratios (HRs) for the effects of interest in mortality models varied over time. However, for the composite of death or mechanical ventilation at 28 days, flexible parametric survival (Royston-Parmar) models were used to allow for time-dependent effects owing to evidence of nonproportional hazards with the Schoenfeld residuals.^20^

Results are presented based on tertiles of admission ferritin values to examine for trends across unbiased categories of ferritin, as there are no clinically relevant thresholds for ferritin elevations in the context of acute inflammation. We opted for tertiles because our cohort was relatively small and a higher number of quantiles (eg, quartiles) would have led to small number of events per group. We supplement our analyses with ferritin examined as a continuous variable, using restricted cubic splines to examine for nonlinear associations with the outcomes, including nonlinear interactions with methylprednisolone use.

The association of methylprednisolone with outcomes was estimated using inverse probability of treatment wights based on the propensity score for methylprednisolone use,^21,22^ a method that has been shown to produce minimal bias among propensity score methods for time-to-event analyses.^21,23,24^ The propensity score was estimated with a logistic regression model that included age, sex, race, ethnicity, smoking, body mass index, hypertension, diabetes, coronary artery disease, atrial fibrillation, congestive heart failure, asthma, chronic lung disease, chronic kidney disease, use of angiotensin-converting enzyme inhibitor or angiotensin receptor blocker, immunocompromised status (ie, >20 mg daily prednisone for ≥1 month, HIV infection, posttransplant immunosuppressive status, or current malignant neoplasm, high-dose chemotherapy, or stem cell transplant within the past year), symptoms duration, oxygen saturation, fraction of inspired oxygen needed at presentation, and admission values of creatinine, C-reactive protein, lymphocyte count, D-dimer, procalcitonin, liver function tests (transaminases and lactic dehydrogenase, as other tests were collinear), and N-terminal pro-B-type natriuretic peptide. We used 15 multiple imputations with chained equations for missing covariate values and combined the estimates.^25,26^ eFigure 1 in the Supplement shows the overlap of the propensity score between the methylprednisolone use groups. The selection of covariates for the propensity score was based on the literature at the time of the study, as data-driven approaches may have had insufficient power in our cohort to detect important associations with the outcomes of interest. Ferritin was not consistently mentioned in the literature as a factor associated with outcomes (and was not associated with outcomes in our cohort); therefore, ferritin level was not included in the propensity score model.

We performed 3 sets of sensitivity analyses. First, we omitted patients who were immunocompromised from the cohort and repeated the analyses. Second, we performed standard (ie, not weighted) adjusted analyses in Cox (for mortality) and Royston-Parmar (for the composite end point) models, using the propensity score as an adjustment variable, since the large number of covariates would cause unstable estimates in enter models. Third, we used falsification end points, ie, outcomes that in principle should have no association with the variables of interest, and rerun the analyses.^27^ Finally, we report E-values for the estimates of methylprednisolone association with outcomes for each ferritin tertile. The E-value quantifies the minimum strength of association on the HR scale that an unmeasured confounder must have with both the treatment and the outcome, while simultaneously considering the measured covariates, to negate the observed treatment-outcome association.^28,29^ The higher the E-value is, the stronger the unmeasured confounding must be to explain the observed association. We used Stata statistical software version 16.1 (StataCorp) for all statistical analyses. *P* values were 2-sided, and statistical significance was set at *P* = .05. Data were analyzed from December 19, 2020, to July 22, 2021.

## Results

### Baseline Characteristics

Among 380 included patients, the median (IQR) age was 60 (49-72) years. A total of 130 patients (34.2%) were women, and 250 patients (65.8%) were men. There were 310 White patients (81.6%), 47 Black patients (12.4%), and 23 Asian patients (6.1%); 131 patients (34.5%) were Hispanic. Median (IQR) ferritin was 936 (490-1585) ng/mL (to convert to micrograms per liter, multiply by 1). The Table summarizes the characteristics of the study population according to admission ferritin tertiles (lower: 29-619 ng/mL; middle: 623-1316 ng/mL; upper: 1322-13 418 ng/mL). Patients with higher ferritin on admission were younger and more likely to be men and less likely to have atrial fibrillation, chronic lung disease, and heart failure; these patients had also higher heart and respiratory rates and lower oxygen saturation, higher creatinine and liver enzyme levels, and higher levels of inflammatory markers, including C-reactive protein, procalcitonin, and interleukin 6 (Table).

**Table.** Baseline Patient Characteristics According to Serum Ferritin Level on Admission

Characteristic	Ferritin tertile, No. (%)		
	Low (29-619 ng/mL) (n = 127)	Middle (623-1316 ng/mL) (n = 127)	High (1322-13 418 ng/mL) (n = 126)
Age, median (IQR), y	65 (49-80)	59 (50-70)	58 (48-67)
Sex			
Men	58 (45.7)	88 (69.3)	104 (82.5)
Female	69 (54.3)	39 (30.7)	22 (17.5)
Race			
White	107 (84.3)	107 (84.3)	96 (76.2)
Black	11 (8.7)	16 (12.6)	20 (15.9)
Asian	9 (7.1)	4 (3.1)	10 (7.9)
Hispanic	39 (30.7)	47 (37.0)	45 (35.7)
BMI, median (IQR)	28.7 (25.6-34.9)	29.6 (26.5-33.3)	29.5 (26.3-32.7)
Comorbidities			
Hypertension	77 (60.6)	69 (54.3)	66 (52.4)
Diabetes	42 (33.1)	38 (29.9)	47 (37.3)
Coronary artery disease	21 (16.5)	14 (11.0)	15 (11.9)
Atrial fibrillation	23 (18.1)	10 (7.9)	8 (6.3)
Chronic lung disease	20 (15.7)	8 (6.3)	8 (6.3)
Chronic kidney disease	10 (7.9)	15 (11.8)	13 (10.3)
Heart failure	17 (13.4)	9 (7.1)	7 (5.6)
Asthma	14 (11.0)	8 (6.3)	7 (5.6)
Immunocompromised	8 (6.3)	14 (11.0)	7 (5.6)
Medication use			
ACE inhibitor	22 (17.3)	18 (14.2)	19 (15.1)
Angiotensin receptor blocker	23 (18.1)	18 (14.2)	17 (13.5)
Statins	49 (38.6)	51 (40.2)	46 (36.5)
Initial vital signs, median (IQR)			
Blood pressure, mm Hg			
Systolic	126 (113-141)	125 (113-138)	124.5 (110-142)
Diastolic	73 (63.5-80.5)	74 (67-82)	74 (67-81)
Heart rate, bpm	98 (83-110)	97.5 (87-109)	102.5 (91-113)
Temperature, °C	38.1 (37.5-39.0)	38.1 (37.4-38.8)	38.1 (37.4-39.2)
Respiratory rate, breath/min	22 (18-28)	22 (18-30)	23 (20-28)
Oxygen saturation, %	91.0 (88.0-94.0)	90.0 (86.0-93.0)	89.5 (86.0-93.0)
Clinical findings, median (IQR)^a^			
Time from symptom onset, d	5 (3-7.5)	7 (5-10)	7 (4-10)
QTc, ms	434 (418-455.5)	433 (416-460)	444 (424-461)
Creatinine, mg/dL	0.9 (0.7-1.1)	1.0 (0.8-1.3)	1.0 (0.8-1.4)
Alanine aminotransferase, U/L	27.0 (17.0-37.0)	38.0 (23.0-55.0)	48.0 (30.0-78.0)
Aspartate aminotransferase, U/L	36.0 (28.0-48.0)	48.0 (31.0-73.0)	63.5 (44.0-97.0)
Lymphocyte count, cells/μL	900 (600-1200)	800 (500-1100)	800 (600-1100)
International normalized ratio	1.2 (1.1-1.3)	1.2 (1.1-1.3)	1.2 (1.1-1.3)
NT-proBNP, pg/mL	297.0 (54.0-1213)	249.0 (41.5-881.5)	145.0 (58.0-943.0)
Troponin, ng/mL	0.0 (0.0-0.0)	0.0 (0.0-0.0)	0.0 (0.0-0.0)
ESR, mm/h	56.0 (28.0-79.0)	50.0 (30.0-79.0)	61.0 (34.5-85.5)
C-reactive protein, mg/dL	9.3 (4.9-15.3)	12.8 (7.1-20.0)	13.6 (7.7-21.8)
D-dimer, ng/mL	339.0 (205.0-711.0)	372.0 (240.0-793.0)	356.5 (265.0-611.0)
Procalcitonin, ng/mL	0.2 (0.1-0.3)	0.2 (0.1-0.4)	0.3 (0.2-0.8)
Lactate dehydrogenase, U/L	331.0 (272.0-445.0)	413.5 (323.0-523.5)	507.5 (396.0-674.0)
Creatine phosphokinase, U/L	113.0 (59.0-259.0)	215.0 (105.0-495.0)	173.0 (93.0-434.0)
Interleukin 6, pg/mL^b^	62.9 (29.8-94.1)	53.0 (17.6-98.2)	71.3 (40.4-116.7)
Concomitant therapies			
Hydroxychloroquine	74 (58.3)	83 (65.4)	55 (43.7)
Azithromycin	57 (44.9)	69 (54.3)	45 (35.7)
Remdesivir	3 (2.4)	2 (1.6)	0
Tocilizumab	24 (18.9)	31 (24.4)	40 (31.7)

Abbreviations: ACE, angiotensin converting enzyme; BMI, body mass index (calculated as weight in kilograms divided by height in meters squared); bpm, beats per minute; ESR, erythrocyte sedimentation rate; NT-proBNP, N-terminal pro-B-type natriuretic peptide; QTc, corrected QT interval on electrocardiogram.

SI conversion factors: To convert ferritin to micrograms per liter, multiply by 1; creatinine to micromoles per liter, multiply by 88.4; alanine aminotransferase and aspartate aminotransferase to microkatals per liter, multiply by 0.0167; lymphocytes to ×10^9^/L, multiply by 0.001; troponin to micrograms per liter, multiply by 1; C-reactive protein to milligrams per liter, multiply by 10; D-dimer to nanomoles per liter, multiply by 5.476; and lactate dehydrogenase to microkatals per liter, multiply by 0.111.

^a^ Findings within 48 hours of admission.

^b^ Available in 81 patients in the low ferritin tertile, 91 patients in the middle ferritin tertile, and 95 patients in the high ferritin tertile.

Compared with patients with available ferritin on admission (380 of 447 patients [85.0%]), those without available ferritin (67 of 447 patients [15.0%]) were older and more likely to have coronary artery disease and atrial fibrillation and had shorter duration of symptoms, higher oxygen saturation on admission, and lower baseline C-reactive protein levels (eTable 1 in the Supplement). Overall, in weighted Cox regression models, patients without ferritin values, compared with those with available ferritin at baseline, had higher 28-day mortality (33.1% vs 21.4%; Cox regression-based test, *P* = .04) but similar 28-day rates of the composite of death or mechanical ventilation (53.8% vs 49.5%; Cox regression-based test, *P* = .74).

### Use of Methylprednisolone

Methylprednisolone was administered in 142 patients (37.4%), including 53 of 126 patients (42.1%) with high ferritin, 46 of 127 patients (36.2%) with ferritin in the middle range, and 43 of 127 patients (33.9%) with low ferritin (*P* = .38). Ferritin levels did not differ between patients who did or did not receive methylprednisolone (median [IQR], 992 [509-1610] ng/mL vs 893 [474-1467] ng/mL; *P* = .32) (eFigure 2 in the Supplement).

Methylprednisolone was initiated a median (IQR) of 2 (1-3) days after admission among patients with high ferritin levels and 2 (1-4) days in those with low ferritin (*P* = .92). This amounted to a median (IQR) of 10 (7-14) days after symptom onset (median [IQR], 11 [8-15] days in patients with high ferritin vs 10 [6-13] in patients with low ferritin; *P* = .16). The daily dose of methylprednisolone did not differ between those with high vs low admission ferritin (median [IQR], 160 [120-240] mg vs 160 [120-180] mg; *P* = .69). Duration of methylprednisolone therapy was a median (IQR) of 5 (4-6) days for patients receiving the full dose (median [IQR], 5 [4-7] days in patients with high ferritin vs 4 [4-6] days in patients with low ferritin; *P* = .21) and 10 (5-14) days for the entire course, including tapering (median [IQR], 10 [6-18] days in patients with high ferritin vs 8 [4-13] days in patients with low ferritin; *P* = .11).

### Methylprednisolone and Clinical Outcomes

A total of 182 patients (47.9%) met the composite outcome of death or mechanical ventilation at 28 days. While 102 patients (26.8%) had received mechanical ventilation and survived, 80 patients (21.1%) died (including 37 patients [9.7%] who died after mechanical ventilation). In weighted analyses, mortality at 28 days was not different between patients who received methylprednisolone vs those who did not (23.0% vs 20.4%; Cox regression-based test, *P* = .58). However, the rate of the composite of death or mechanical ventilation at 28 days was lower among those who received methylprednisolone vs those who did not (45.3% vs 51.9%; Cox-regression-based test, *P* = .03).

Using patients with ferritin levels in the lower tertile as the reference group, patients with ferritin levels in the middle and high ferritin tertiles did not have higher risk of 28-day mortality (middle: HR, 0.97; 95% CI, 0.56-1.68; high: HR, 0.87; 95% CI, 0.49-1.57) or the composite end point (middle: HR, 1.14; 95% CI, 0.80-1.63; high: HR, 1.15; 95% CI, 0.79-1.66 ) in weighted Cox regression models. Evaluation with restricted cubic splines did not find any nonlinear association of admission ferritin level with outcomes.

### Ferritin, Methylprednisolone, and Clinical Outcomes

**Figure 2.**
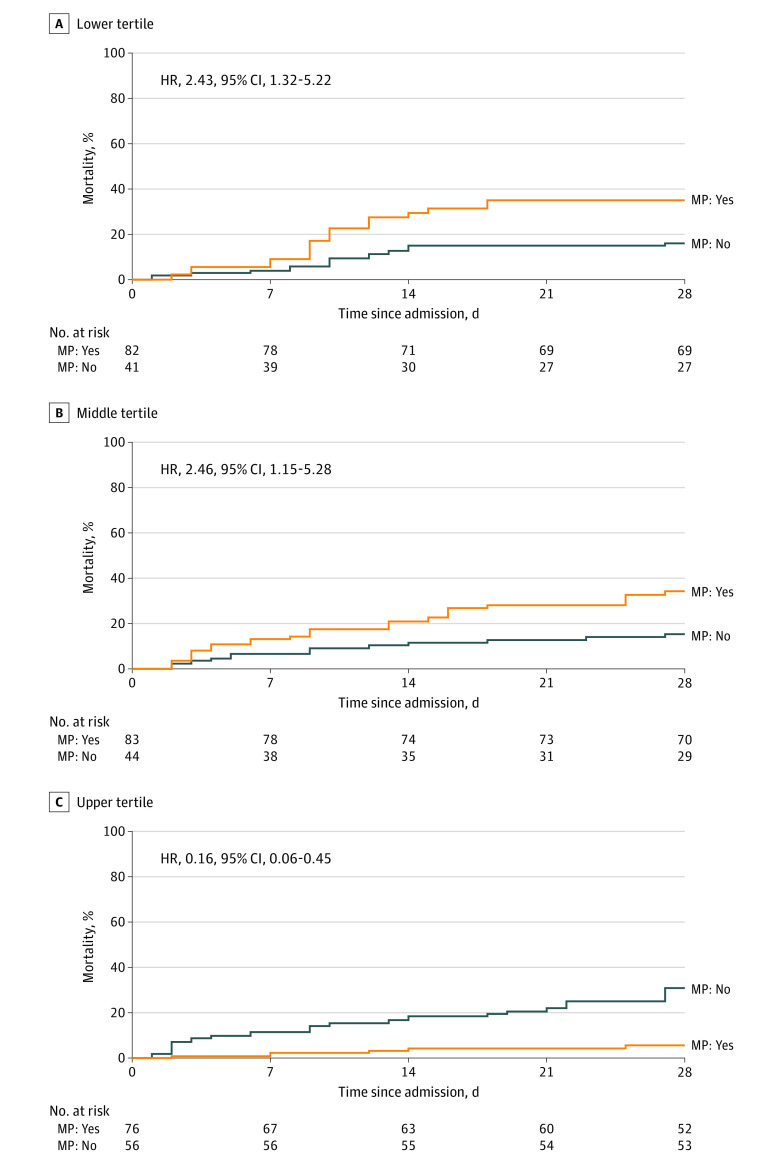
Cumulative Incidence of Mortality According to Ferritin Tertile on Admission and Use of Methylprednisolone (MP) Estimates are weighted by the inverse probability of MP treatment (based on a propensity score).

In Cox regression models weighted by the inverse probability of methylprednisolone use (based on a propensity score), methylprednisolone use was associated with higher mortality in patients with admission ferritin in the lower tertile (HR, 2.43; 95% CI, 1.13-5.22) or the middle tertile (HR, 2.46; 95% CI, 1.15-5.28) but lower mortality among those with ferritin in the upper tertile (HR, 0.16; 95% CI, 0.06-0.45; *P* for interaction < .001) (Figure 2). The full model is presented in the eAppendix in the Supplement.

**Figure 3.**
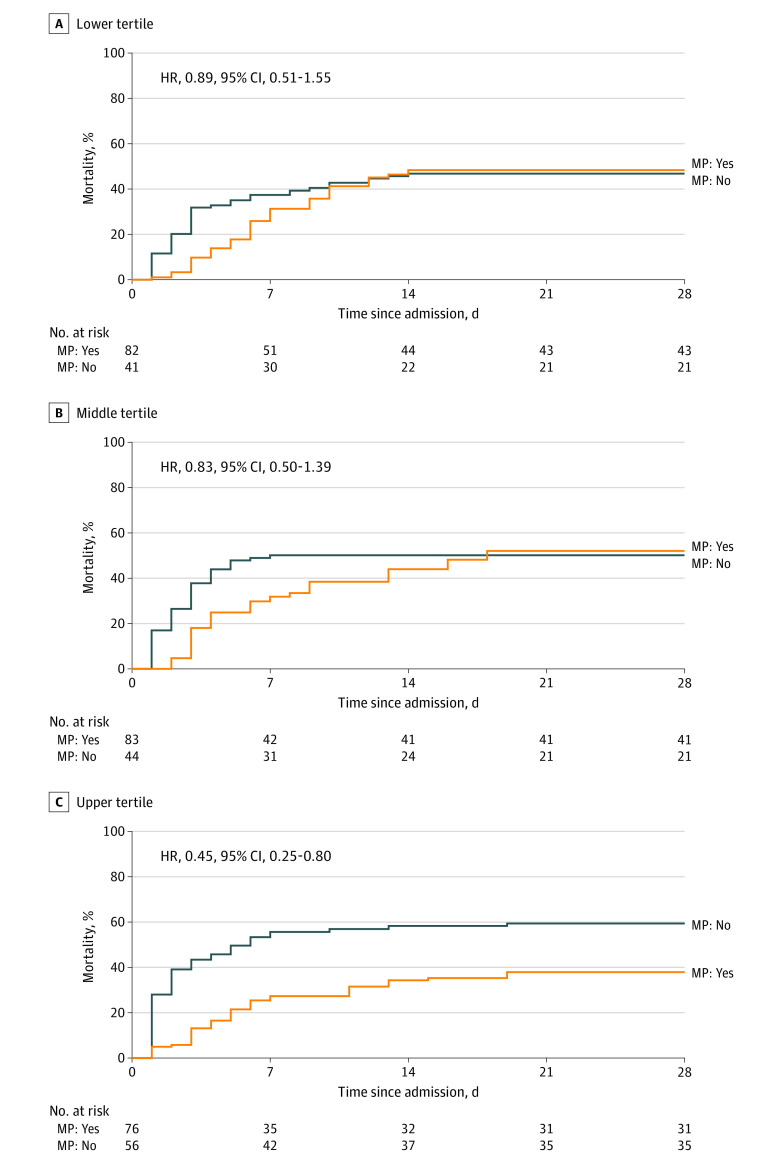
Cumulative Incidence of Death or Mechanical Ventilation According to Ferritin Tertile on Admission and Use of Methylprednisolone (MP) Estimates are weighted by the inverse probability of MP treatment (based on a propensity score).

In weighted Royston-Parmar models (to accommodate for nonproportional hazards of methylprednisolone), methylprednisolone was not associated with the composite of death or mechanical ventilation in patients with ferritin in the lower tertile (HR, 0.89; 95% CI, 0.51-1.55) or the middle tertile (HR, 0.83; 95% CI, 0.50-1.39); however, there was an association with lower rates of the composite end point in patients with ferritin in the upper tertile (HR, 0.45; 95% CI, 0.25-0.80; *P* for interaction = .11) (Figure 3). The full model is presented in the eAppendix in the Supplement. Analyses with ferritin treated as a continuous variable, evaluated with restricted cubic splines for nonlinear associations, also suggested an interaction between ferritin level and methylprednisolone on outcomes (eFigure 3 and eFigure 4 in the Supplement).

### Sensitivity Analyses

The results for mortality did not change substantially when patients who were immunocompromised were removed from the analysis (eTable 2 in the Supplement); however, for the composite end point, methylprednisolone remained significantly associated with benefit only in the upper tertile of ferritin values, but the interaction was attenuated. In standard regression models (ie, not weighted by inverse probability of treatment) adjusted for the propensity score, the differential association with mortality persisted (eTable 3 in the Supplement). However, the interaction for the composite end point was attenuated, with HRs indicating a gradient for benefit across increasing ferritin tertiles. The full model for this analysis is presented in the eAppendix in the Supplement. In models with 2 different falsification end points as outcomes, there was no differential association of methylprednisolone with these end points (eTable 4 in the Supplement). The E-values for the association of methylprednisolone with outcomes across ferritin levels are presented in eTable 5 in the Supplement. For the upper ferritin tertile, the association with reduced mortality could be explained away by an unmeasured confounder associated with both the treatment and the outcome by a risk ratio of 11.98; the corresponding risk ratio required to explain away the association with the composite end point would be 3.87.

## Discussion

In this single-center cohort study, administration of methylprednisolone in nonintubated patients with COVID-19 receiving high-flow oxygen therapy was associated with clinical benefit only in patients with admission ferritin levels in the upper tertile of values. In this subgroup, methylprednisolone was associated with approximately 80% lower mortality and more than 50% lower risk of the composite end point of death or mechanical ventilation at 28 days. In contrast, in patients with serum ferritin in the lower and middle tertiles of values on admission, methylprednisolone use was not associated with benefit in mortality or the 28-day composite end point. The observed differential association of methylprednisolone use with outcomes raises the question whether a biomarker-based patient selection for corticosteroid use in severe COVID-19 should be investigated.

Currently, in view of limited supporting data, the selection of patients who might benefit from corticosteroids has been left to the discretion of the treating physicians, even in the context of randomized clinical trials.^3^ Corticosteroids have been proven to be beneficial in severe COVID-19^3^ during the intense inflammatory process that follows the initial viral replication phase.^30,31^ However, the use of corticosteroids comes with a cost. In prior coronavirus outbreaks, ie, severe acute respiratory syndrome and Middle East respiratory syndrome, impaired virus clearance with corticosteroid use has been reported.^32,33^ In addition, the emerging use of monoclonal antibodies,^34^ in which the risk of synergistic immunosuppression may outweigh the benefits, makes risk stratification for corticosteroid use imperative.

Inflammatory markers have been widely used to detect disease progression from viral invasion and replication^31^ to intense inflammatory responses and cytokine storm.^35^ Ferritin has been included in clinically used inflammatory marker panels, but little is known about its cellular uptake or release and its downstream fate after endocytosis.^36^ Whether ferritin is a pro-inflammatory marker, an anti-inflammatory marker, or a byproduct of damaged cells released during an infection is unknown. There are 3 main regulatory pathways for the expression of ferritin,^36^ driven by increased serum iron, hypoxia, and inflammation. During inflammation, ferritin may be released from macrophages or cells owing to tissue damage and may be regulated by tumor necrosis factor α, interleukin 2, and interleukin 10. Ferritin also may have a role as an anti-inflammatory mediator by binding to high molecular–weight kininogen and inhibiting its cleavage, resulting in reduced bradykinin release and a decrease in the total inflammatory response.^36^ Cytosolic ferritin is composed of 24 subunits of either the L- or H-type chains. In the cell, H-ferritin offers the host tolerance to infections and sepsis, possibly through cytosolic iron sequestration and overall control of oxidative stress. For example, H-ferritin facilitates cell adaptation to malaria, and inhibition of H-ferritin leads to increased cell susceptibility with elevated oxidative stress and tissue damage.^17^ Similarly, the activity of H-ferritin on cytosolic iron sequestration is critical for cell tolerance to sepsis.^16^ In the same direction, H-ferritin knockout mice experience increased inflammatory response, higher bacterial load, and reduced survival after infection with mycobacterium tuberculosis.^18^

While several studies have reported that elevated ferritin levels are associated with worse outcomes in COVID-19, there are no data on nonlinear associations with outcomes or interactions with immunomodulatory therapies for outcomes. Our observations suggest that serum ferritin levels may reflect the balance between beneficial host immune responses vs deleterious inflammatory overresponse in patients with COVID-19 pneumonia. Therefore, although serum levels up to 1300 ng/mL (the upper tertile cutoff in our study) would be outside reference ranges in the context of an elective evaluation, this degree of ferritin elevation may reflect a healthy host response to an acute infection like COVID-19, indicating activation of a beneficial defensive mechanism. As corticosteroids act broadly, inhibiting multiple pathways in the inflammatory process,^37^ suppressing this moderate, healthy response may lead to worse outcomes. Conversely, ferritin elevations beyond this level (>1300 ng/mL) may reflect a dysregulated, harmful inflammatory overresponse where corticosteroids can help.^38^ Therefore, the possibility that corticosteroids may have dramatically different effects depending on the degree of host immune response, especially in the context of adjunct immunomodulatory therapies (eg, monoclonal antibodies), needs further investigation.

### Limitations

Our study has several limitations. First, despite using a propensity score–based method (inverse probability of treatment weighting) to minimize bias for corticosteroid use, it is likely that there is still residual confounding by indication and other unobserved confounders. Second, ferritin levels were available in 85% of potentially eligible patients during the cohort inception period, and this may have introduced bias. Third, despite the high event rate in this high-risk COVID-19 cohort, the sample size and thus the absolute number of events was relatively small, and this may have led to unstable estimates in subgroups. This is likely the reason for the inconsistent findings regarding the differential association of methylprednisolone with mortality vs the composite of death or mechanical ventilation in the ferritin subgroups. Fourth, we studied the differential outcomes of methylprednisolone treatment in association with ferritin levels on presentation among patients receiving high-flow oxygen; therefore, our results may not apply to milder forms of COVID-19. Fifth, the use and timing of other therapies for COVID-19 has changed substantially over time, as has the case mix of patients admitted to hospitals with COVID-19, compared with the early waves of the pandemic. Therefore, our findings may not be generalizable to patients currently being admitted with COVID-19.

## Conclusions

In this cohort study, methylprednisolone in nonintubated patients with COVID-19 receiving high-flow oxygen therapy was associated with clinical benefit only in patients with admission ferritin levels in the upper tertile of ferritin values. In contrast, for patients with ferritin in the lower and middle tertiles, no association with benefit was observed. Our findings warrant prospective investigation, especially in view of adjunct immunomodulatory therapies that are increasingly being used and tested in patients with COVID-19.

## Notes

Supplement. **eAppendix.** Additional Results **eTable 1.** Baseline Patient Characteristics According to Availability of Ferritin at Baseline **eTable 2.** Association of Methylprednisolone Therapy With Outcomes in Patients Without Immunocompromised Status at Baseline **eTable 3.** Association of Methylprednisolone Therapy With Outcomes According to Serum Ferritin at Baseline in Standard Regression Models Adjusting for the Propensity Score **eTable 4.** Association of Methylprednisolone Therapy With Falsification End Points According to Ferritin Tertiles at Baseline **eTable 5.** E-values for the Association of Methylprednisolone Therapy With End Points According to Ferritin Tertiles at Baseline **eFigure 1.** Distribution of the Propensity Score Between the Methylprednisolone Use Groups **eFigure 2.** Distribution of Admission Ferritin Levels According to Use of Methylprednisolone **eFigure 3.** Interaction of Continuous Ferritin With Methylprednisolone Use for Mortality **eFigure 4.** Interaction of Continuous Ferritin With Methylprednisolone Use for the Composite of Death or Mechanical Ventilation
