# Supplementary material for: Association of Serum Ferritin Levels and Methylprednisolone Treatment With Outcomes in Nonintubated Patients With Severe COVID-19 Pneumonia
Source: JAMA Netw Open. 2021 Oct 4;4(10):e2127172. doi: 10.1001/jamanetworkopen.2021.27172 (PMC8491101; doi:10.1001/jamanetworkopen.2021.27172)
Supplement: Supplement. — eAppendix. Additional Results eTable 1. Baseline Patient Characteristics According to Availability of Ferritin at Baseline eTable 2. Association of Methylprednisolone Therapy With Outcomes in Patients Without Immunocompromised Status at Baseline eTable 3. Association of Methylprednisolone Therapy With Outcomes According to Serum Ferritin at Baseline in Standard Regression Models Adjusting for the Propensity Score eTable 4. Association of Methylprednisolone Therapy With Falsification End Points According to Ferritin Tertiles at Baseline eTable 5. E-values for the Association of Methylprednisolone Therapy With End Points According to Ferritin Tertiles at Baseline eFigure 1. Distribution of the Propensity Score Between the Methylprednisolone Use Groups eFigure 2. Distribution of Admission Ferritin Levels According to Use of Methylprednisolone eFigure 3. Interaction of Continuous Ferritin With Methylprednisolone Use for Mortality eFigure 4. Interaction of Continuous Ferritin With Methylprednisolone Use for the Composite of Death or Mechanical Ventilation [file jamanetwopen-e2127172-s001.pdf]

Supplemental Online Content

Papamanoli A, Kalogeropoulos AP, Hotelling J, et al. Association of serum ferritin levels and methylprednisolone treatment with outcomes in nonintubated patients with severe COVID-19 pneumonia. *JAMA Netw Open*. 2021;4(10):e2127172. doi:10.1001/jamanetworkopen.2021.27172

eAppendix. Additional Results
eTable 1. Baseline Patient Characteristics According to Availability of Ferritin at Baseline
eTable 2. Association of Methylprednisolone Therapy With Outcomes in Patients Without Immunocompromised Status at Baseline
eTable 3. Association of Methylprednisolone Therapy With Outcomes According to Serum Ferritin at Baseline in Standard Regression Models Adjusting for the Propensity Score
eTable 4. Association of Methylprednisolone Therapy With Falsification End Points According to Ferritin Tertiles at Baseline
eTable 5. E-values for the Association of Methylprednisolone Therapy With End Points According to Ferritin Tertiles at Baseline
eFigure 1. Distribution of the Propensity Score Between the Methylprednisolone Use Groups
eFigure 2. Distribution of Admission Ferritin Levels According to Use of Methylprednisolone
eFigure 3. Interaction of Continuous Ferritin With Methylprednisolone Use for Mortality
eFigure 4. Interaction of Continuous Ferritin With Methylprednisolone Use for the Composite of Death or Mechanical Ventilation

This supplemental material has been provided by the authors to give readers additional information about their work.

© 2021 Papamanoli A et al. *JAMA Network Open.*

eAppendix. Additional Results

Ferritin, Methylprednisolone, and Clinical Outcomes

The full Cox regression model for the interaction been ferritin tertiles and methylprednisolone for mortality is presented below. The estimates are weighted by the inverse probability of treatment with methylprednisolone based on a propensity score. The estimates for each ferritin tertile that appear in the main manuscript have been calculated using linear combination of estimates.

Effect	HR (95%CI)	P value
Methylprednisolone	2.45 (1.13-5.31)	0.023
Ferritin		
Lower tertile	Reference	-
Middle tertile	0.95 (0.44-2.07)	0.90
Upper tertile	2.05 (1.01-4.18)	0.047
MP x Ferritin		
Lower tertile	Reference	-
Middle tertile	1.01 (0.34-2.99)	0.99
Upper tertile	0.07 (0.02-0.24)	<0.001

MP: Methylprednisolone

The full Royston-Parmar regression model for the interaction been ferritin tertiles and methylprednisolone for the composite of death or mechanical ventilation is presented below. The estimates are weighted by the inverse probability of treatment with methylprednisolone based on a propensity score. The estimates for each ferritin tertile that appear in the main manuscript have been calculated using linear combination of estimates.

Effect	HR (95%CI)	P value
Methylprednisolone	0.57 (0.32-1.03)	0.061
Ferritin		
Lower tertile	Reference	-
Middle tertile	1.18 (0.74-1.88)	0.49
Upper tertile	1.55 (0.97-2.48)	0.068
MP x Ferritin		
Lower tertile	Reference	-
Middle tertile	0.96 (0.44-2.07)	0.91
Upper tertile	0.49 (0.21-1.11)	0.086
RCS1	1.90 (1.75-2.06)	<0.001
RCS2	1.37 (1.31-1.43)	<0.001
MPxRCS1	2.32 (1.49-3.62)	<0.001
MPxRCS2	1.31 (1.00-1.71)	0.047
Constant	0.40 (0.29-0.56)	<0.001

MP: Methylprednisolone; RCS1: First restricted cubic spline term used to model time; RCS2: Second restricted cubic spline term used to model time

© 2021 Papamanoli A et al. *JAMA Network Open.*

Sensitivity Analyses

The Cox model using standard (i.e., not weighted) regression analyses for the interaction between ferritin tertiles and methylprednisolone use, adjusted for the propensity score, is presented below. The estimates for each ferritin tertile that appear in **eTable 3** have been calculated using linear combination of estimates.

Effect	HR (95%CI)	P value
Methylprednisolone	1.92 (0.91-4.08)	0.089
Ferritin		
Lower tertile	Reference	-
Middle tertile	0.91 (0.42-1.95)	0.80
Upper tertile	1.48 (0.74-2.95)	0.26
MP x Ferritin		
Lower tertile	Reference	-
Middle tertile	0.98 (0.34-2.80)	0.97
Upper tertile	0.16 (0.05-0.53)	0.003
Propensity score	4.20 (1.05-16.7)	0.042

MP: Methylprednisolone

The Royston-Parmar model using standard (i.e., not weighted) regression for the interaction between ferritin tertiles and methylprednisolone for the composite of death or mechanical ventilation is presented below. The estimates for each ferritin tertile that appear in eTable 3 have been calculated using linear combination of estimates.

Effect	HR (95%CI)	P value
Methylprednisolone	0.56 (0.31-1.01)	0.054
Ferritin		
Lower tertile	Reference	-
Middle tertile	1.21 (0.77-1.90)	0.417
Upper tertile	1.46 (0.93-2.30)	0.098
MP x Ferritin		
Lower tertile	Reference	-
Middle tertile	0.84 (0.40-1.77)	0.653
Upper tertile	0.58 (0.27-1.22)	0.153
Propensity score		
RCS1	5.06 (2.00-12.8)	0.001
RCS2	1.98 (1.77-2.22)	0.000
MPxRCS1	1.39 (1.30-1.48)	0.000
MPxRCS2	2.05 (1.42-2.95)	0.000
Constant	1.28 (1.03-1.59)	0.028

MP: Methylprednisolone; RCS1: First restricted cubic spline term used to model time; RCS2: Second restricted cubic spline term used to model time

© 2021 Papamanoli A et al. *JAMA Network Open.*

eTable 1. Baseline Patient Characteristics According to Availability of Ferritin at Baseline

Characteristic	Ferritin Available (N=380)	Ferritin Unavailable (N=67)	*P* value*
**Demographics**			
Age, years	60 (49 – 72)	63 (54 – 80)	0.015
Female, N (%)	130 (34.2)	26 (38.8)	0.489
Race, N (%)			0.620
White	310 (81.6)	53 (79.1)	
Black	47 (12.4)	9 (13.4)	
Asian	23 (6.1)	5 (7.5)	
Hispanic, N (%)	131 (34.5)	19 (28.4)	0.400
Body mass index, kg/m^2	29.3 (26.1 – 33.5)	29.5 (25.7 – 35.6)	0.570
**Comorbidities, N (%)**			
Hypertension	212 (55.8)	42 (62.7)	0.349
Diabetes	127 (33.4)	20 (29.9)	0.672
Coronary artery disease	50 (13.2)	17 (25.4)	0.015
Atrial fibrillation	41 (10.8)	16 (23.9)	0.005
Chronic lung disease	36 (9.5)	12 (17.9)	0.052
Chronic kidney disease	38 (10)	8 (11.9)	0.662
Heart failure	33 (8.7)	9 (13.4)	0.253
Asthma	29 (7.6)	2 (3)	0.202
Immunocompromised	29 (7.6)	4 (6)	0.802
**Medication Use, N (%)**			
ACE inhibitor	59 (15.5)	10 (14.9)	1.000
Angiotensin receptor blocker	58 (15.3)	15 (22.4)	0.153
Statins	146 (38.4)	28 (41.8)	0.684
**Initial Vital Signs, N (%)**			
Systolic blood pressure, mmHg	125 (111-141)	127 (114-144.5)	0.752
Diastolic blood pressure, mmHg	74 (66-81)	72 (60-79)	0.128
Heart rate, bpm	100 (86-111)	99 (87-110.5)	0.946
Temperature, °C	38.1 (37.5-39)	38.3 (37.6-39.1)	0.641
Respiratory rate, bpm	22 (18-28)	20 (18-25)	0.097
Oxygen saturation (%)	90 (87-93)	92 (89-94)	0.010

© 2021 Papamanoli A et al. *JAMA Network Open.*

**Clinical Findings†, N (%)**			
Symptom duration on presentation, days	7 (3.5-9)	5 (2-8)	0.041
QTc, ms	437 (418-459)	442 (419-471)	0.172
Creatinine, mg/dL	0.96 (0.76-1.27)	1.02 (0.83-1.40)	0.094
Alanine aminotransferase, IU/L	35 (22-56)	29 (18-47.5)	0.050
Aspartate aminotransferase, IU/L	46 (32-72)	42.5 (29-60)	0.154
Lymphocyte count, K/uL	0.82 (0.57-1.12)	0.90 (0.61-1.21)	0.227
International normalized ratio	1.2 (1.1-1.3)	1.2 (1.1-1.3)	0.543
NT-proBNP, pg/mL	185 (50-981)	281 (80-1408)	0.312
Troponin, ng/mL	0.01 (0.01-0.01)	0.01 (0.01-0.01)	0.949
ESR, mm/h	55 (31-80)	48.5 (23-69)	0.196
C-reactive protein, mg/dL	12.1 (6.6-19.6)	9.5 (4.5-15.3)	0.019
D-dimer, ng/mL	356.6 (238-731)	364.5 (281-790)	0.410
Procalcitonin, ng/mL	0.21 (0.13-0.49)	0.19 (0.12-0.45)	0.395
Lactate dehydrogenase, IU/L	415 (307-538)	355 (270-514)	0.075
Creatine phosphokinase, IU/L	163 (81-384)	125 (58.5-244.5)	0.220
Interleukin-6, pg/mL ‡	63.4 (29.8-102)	70.8 (32.5-138.5)	0.335
**Concomitant Therapies, N (%)**			
Hydroxychloroquine	212 (55.8)	48 (71.6)	0.016
Azithromycin	171 (45)	43 (64.2)	0.005
Remdesivir	5 (1.3)	1 (1.5)	1.000
Tocilizumab	95 (25)	13 (19.4)	0.357

Values are N (%) or median (25th, 75th percentile).

* Mann-Whitney test for continuous variables and Fisher's exact test for categorical variables.

† Findings within 48 hours of admission

‡ Available in 267 and 46 patients, respectively

ACE: angiotensin converting enzyme; ESR: erythrocyte sedimentation rate; NT-proBNP: N-terminal pro-B-type natriuretic peptide; QTc: corrected QT interval on electrocardiogram

© 2021 Papamanoli A et al. *JAMA Network Open.*

eTable 2. Association of Methylprednisolone Therapy With Outcomes in Patients Without Immunocompromised Status at Baseline

	28-day mortality	28-day death or mechanical ventilation
	**Hazard ratio (95%CI)**	**Hazard Ratio (95%CI)**
**Ferritin tertile 1**	2.30 (1.05–5.04)	0.95 (0.54–1.68)
**Ferritin tertile 2**	2.56 (1.18–5.55)	0.89 (0.52–1.52)
**Ferritin tertile 3**	0.21 (0.07–0.59)	0.57 (0.33–0.98)
***P* for interaction** †	<0.001	0.23

* Defined as >20 mg daily prednisone for ≥1 month; human immunodeficiency virus infection; post-transplant immunosuppressive status; current malignancy, high dose chemotherapy, or stem cell transplant within the past year.
† Joint interaction of ferritin tertile with methylprednisolone use.
Estimates for mortality calculated with Cox regression models and for death or mechanical ventilation calculated with Royston-Parmar models (because of non-proportional hazards).

eTable 3. Association of Methylprednisolone Therapy With Outcomes According to Serum Ferritin at Baseline in Standard Regression Models Adjusting for the Propensity Score

	28-day mortality	28-day death or mechanical ventilation
	**Hazard ratio (95%CI)**	**Hazard Ratio (95%CI)**
**Ferritin tertile 1**	1.92 (0.91–4.08)	0.80 (0.46–1.40)
**Ferritin tertile 2**	1.89 (0.87–4.09)	0.67 (0.40–1.14)
**Ferritin tertile 3**	0.30 (0.11–0.80)	0.46 (0.27–0.79)
***P* for interaction ***	0.004	0.32

† Joint interaction of ferritin tertile with methylprednisolone use. Estimates for mortality calculated with Cox regression models and for death or mechanical ventilation calculated with Royston-Parmar models (because of non-proportional hazards).

© 2021 Papamanoli A et al. *JAMA Network Open.*

eTable 4. Association of Methylprednisolone Therapy With Falsification End Points According to Ferritin Tertiles at Baseline

	Falsification Endpoint 1 *	Falsification Endpoint 2 †
	**Odds Ratio (95%CI)**	**Odds Ratio (95%CI)**
**Ferritin tertile 1**	1.21 (0.58–2.52)	0.75 (0.36–1.58)
**Ferritin tertile 2**	0.82 (0.40–1.69)	1.15 (0.55–2.37)
**Ferritin tertile 3**	0.85 (0.42–1.73)	0.74 (0.36–1.51)
***P* for interaction ‡**	0.33	0.36

* Falsification endpoint 1: even (1) vs. odd (0) patient age on admission (treated as an integer). † Falsification endpoint 2: even (1) or odd (0) date of discharge (or death) in computer format (e.g., March 23, 2020, is 21997). ‡ Joint interaction of ferritin tertile with methylprednisolone use. Estimates calculated with logistic regression weighted by the inverse probability of treatment with methylprednisolone using the propensity score as described in the main manuscript.

eTable 5. E-values for the Association of Methylprednisolone Therapy With End Points According to Ferritin Tertiles at Baseline

	28-day mortality	28-day death or mechanical ventilation
**Ferritin tertile 1**	4.29	1.50
**Ferritin tertile 2**	4.36	1.70
**Ferritin tertile 3**	12.0	3.87

E-values calculated with the *evalue* module for STATA written by Linden, Mathur, and van der Weele, using the *common* option (i.e., outcome incidence is between 15% and 85%). E-value is defined as the minimum strength of association on the risk ratio scale that an unmeasured confounder would need to have with both the treatment and the outcome to fully explain away a specific treatment-outcome association, conditional on the measured covariates.

© 2021 Papamanoli A et al. *JAMA Network Open.*

eFigure 1. Distribution of the Propensity Score Between the Methylprednisolone Use Groups

The propensity score was estimated with a logistic regression model that included age, sex, race, ethnicity, smoking, body mass index, hypertension, diabetes, coronary artery disease, atrial fibrillation, congestive heart failure, asthma, chronic lung disease, chronic kidney disease, use of angiotensin-converting enzyme inhibitor or angiotensin receptor blocker, immuno-compromised status (>20 mg daily prednisone for ≥1 month; human immunodeficiency virus infection; post-transplant immunosuppressive status; current malignancy, high dose chemotherapy, or stem cell transplant within the past year), symptoms duration, oxygen saturation, FiO2 needed at presentation, and admission values of creatinine, C-reactive protein, lymphocyte count, D-dimer, procalcitonin, liver function tests (transaminases and lactic dehydro-genase as other tests were collinear), and N-terminal pro-B-type natriuretic peptide. We used multiple imputations (N=15) with chained equations for missing covariate values and combined estimates. Lines represent median (orange), 25th percentile (red), and 75th percentile (green).

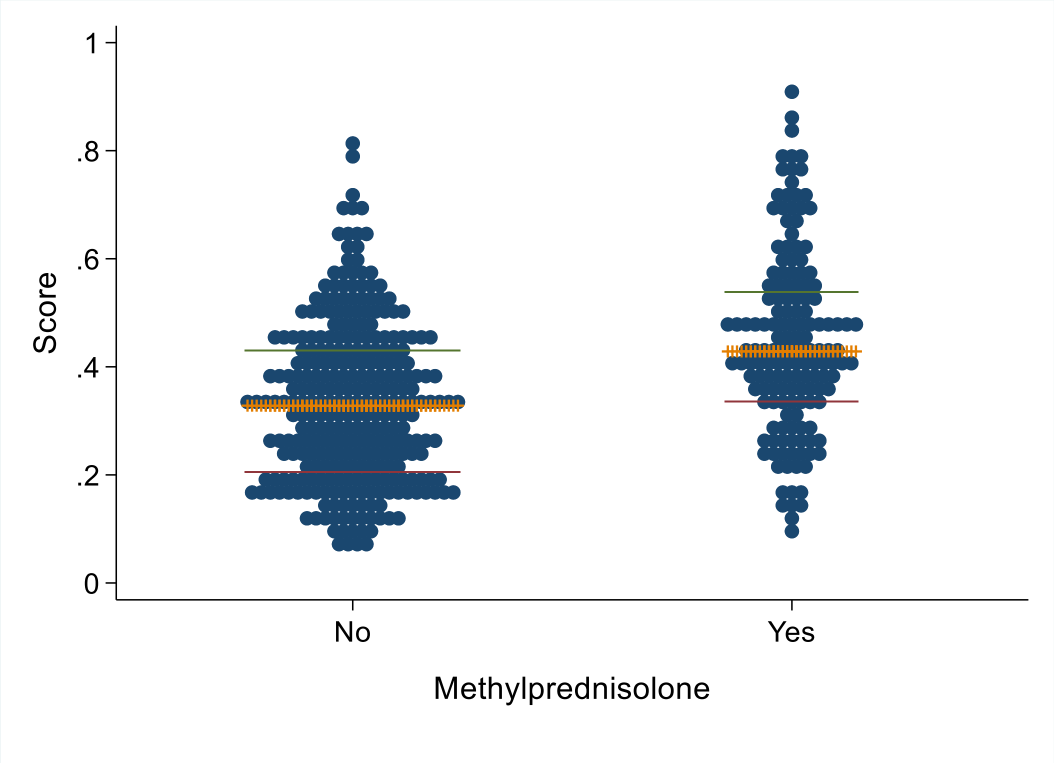

© 2021 Papamanoli A et al. *JAMA Network Open.*

eFigure 2. Distribution of Admission Ferritin Levels According to Use of Methylprednisolone

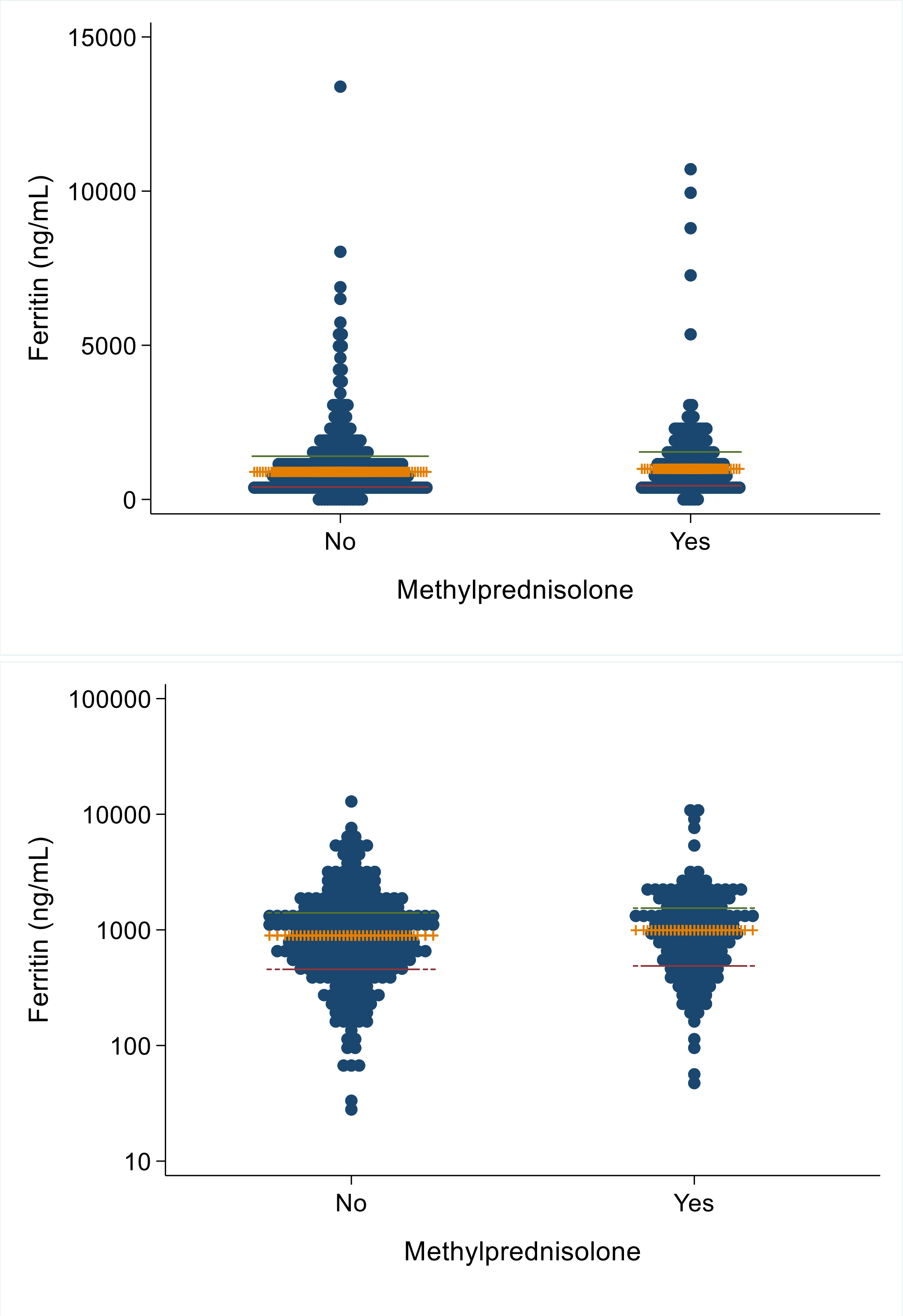

Natural (upper panel) and log-scale (lower panel) axis. Lines represent median (orange), 25th percentile (red), and 75th percentile (green)

© 2021 Papamanoli A et al. *JAMA Network Open.*

eFigure 3. Interaction of Continuous Ferritin With Methylprednisolone Use for Mortality

The association of untransformed ferritin with 28-day mortality was modeled with restricted cubic splines (to allow for detection of nonlinear forms of association), in a standard Cox model (as there was no evidence against proportionality) including ferritin, methylprednisolone use and the interaction term of ferritin with methylprednisolone (as a continuous variable). We allowed for 4 degrees of freedom for each continuous variable — and the algorithm used 2. The log-hazard (linear predictor) lines for the association of methylprednisolone with mortality crossed at approximately 1000 ng/mL. Note: the x axis is in log scale to facilitate visual assessment.

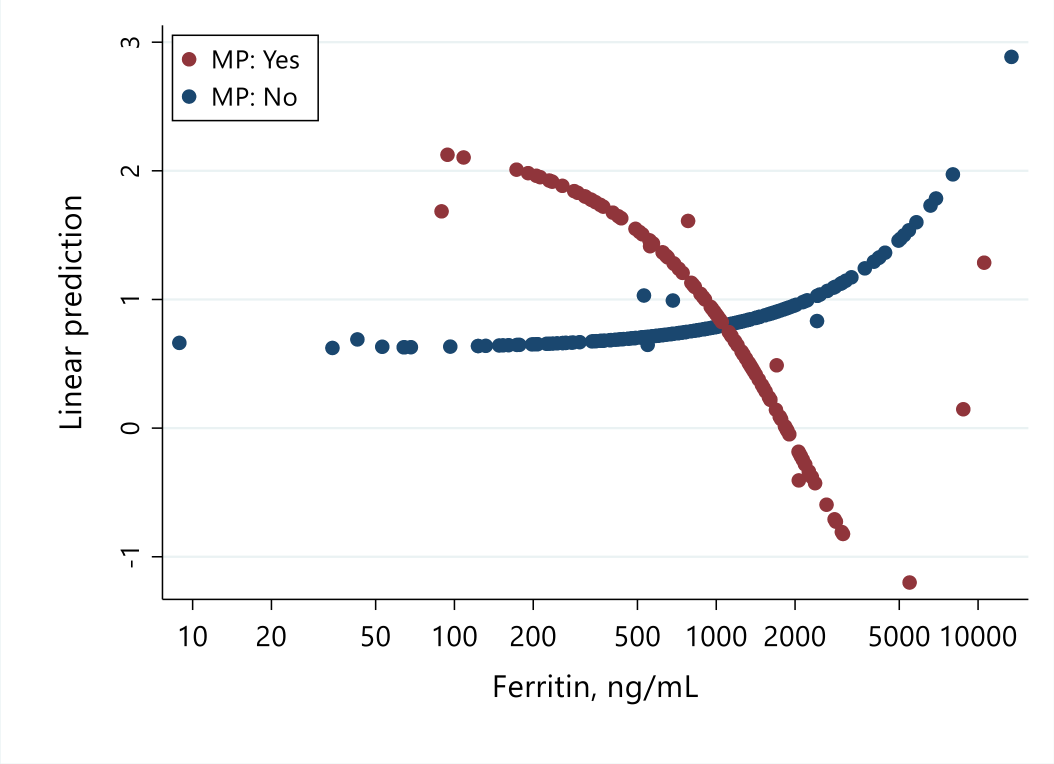

© 2021 Papamanoli A et al. *JAMA Network Open.*

eFigure 4. Interaction of Continuous Ferritin With Methylprednisolone Use for the Composite of Death or Mechanical Ventilation

The association of untransformed ferritin with the 28-day composite of death or mechanical ventilation mortality was modeled with restricted cubic splines (to allow for detection of nonlinear forms of association), in a Royston-Parmar model (as there was evidence of non-proportionality for the methylprednisolone effect in standard Cox models) including ferritin, methylprednisolone use and the interaction term of ferritin with methylprednisolone (as a continuous variable). We plotted polynomial fit lines for the log-hazard (linear predictor) to facilitate interpretation. The lines for the association of methylprednisolone with the composite endpoint separated before approximately 1000 ng/mL. Note: the x axis is in log scale to facilitate visual assessment.

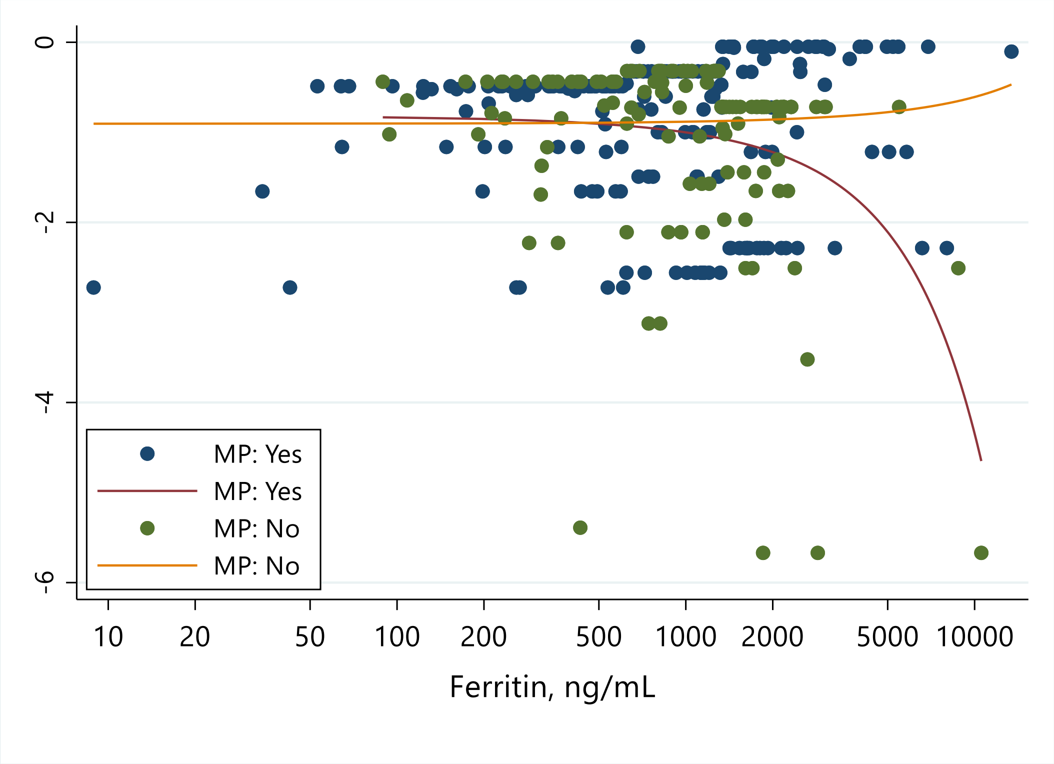

© 2021 Papamanoli A et al. *JAMA Network Open.*
